# Exercise, daily physical activity, and age-specific cumulative incidence of mobility decline: an 8-year cohort study of locomotive syndrome

**DOI:** 10.1186/s11556-026-00424-y

**Published:** 2026-06-29

**Authors:** Satoshi Yamaguchi, Keiko Yamada, Masaru Moriyama, Tomoyuki Arai, Yasuhiro Morita, Hideaki Ishibashi, Tadashi Ohe, Seiji Ohtori, Ryo Nakagawa

**Affiliations:** 1 Graduate School of Global and Transdisciplinary Studies, Chiba University, Chiba, Japan; 2 Department of Liberal Arts, Faculty of Healthcare and Welfare, Saitama Prefectural University, Koshigaya, Japan; 3 Omiya City Clinic, Omiya, Japan; 4 Department of Physical Therapy Faculty of Health and Medical Care, Saitama Medical University, Morohongō, Japan; 5 Department of Orthopedic Surgery, Ina Hospital, Saitama, Japan; 6 Department of Orthopaedic Surgery, NTT Medical Center, Tokyo, Japan; 7 Department of Orthopaedic Surgery, Graduate School of Medical and Pharmaceutical Sciences, Chiba University, Chiba, Japan

**Keywords:** Mobility decline, Locomotive syndrome, Physical activity, Exercise habits

## Abstract

**Background:**

Mobility decline is a gradual process that begins earlier than recognized. Locomotive syndrome (LS) captures early mobility impairment using performance tests and a self-reported questionnaire. Although physical activity is important for maintaining mobility, whether simple self-reported exercise habits and daily activity relate to long-term age-specific risk of incident LS has not been fully investigated. This study examined age-specific associations of exercise habits and daily activity with the eight-year cumulative incidence of LS.

**Methods:**

This cohort study included 21,339 Japanese adults aged 21–84 (median, 48) years without LS at baseline who participated in annual health checkups between 2016 and 2024. Baseline exercise habits and daily activity were assessed by self-report using dichotomous questionnaire items, and participants were classified into four groups according to the combination of these responses: inactive (Ex−/Da−), daily activity only (Ex−/Da+), exercise only (Ex+/Da−), and both exercise and daily activity (Ex+/Da+). Analyses were stratified by age (≤ 39, 40–64, ≥ 65 years). Incident LS was defined based on the two-step test, stand-up test, and 25-item Geriatric Locomotive Function Scale. Cumulative incidence was estimated using Kaplan–Meier methods, and associations were assessed with multivariable Cox proportional hazards models adjusted for lifestyle-related factors.

**Results:**

Over eight years, 7183 participants developed incident LS. Among adults aged 40–64 years, cumulative LS incidence at 8 years showed a statistically significant decline from 0.45 in the Ex−/Da− group to 0.37 in the Ex+/Da+ group (log-rank *P* < 0.001), which showed the lowest adjusted risk (hazard ratio, 0.79, 95% CI, 0.72–0.85). A similar statistically significant association was observed in adults aged ≥ 65 years (log-rank *P* < 0.001), with the lowest adjusted risk in the Ex+/Da+ group (hazard ratio, 0.70, 95% CI, 0.56–0.86). The incidence was higher when LS was defined by the 25-item Geriatric Locomotive Function Scale, suggesting that subjective functional decline precedes performance-based impairment.

**Conclusion:**

Mobility decline, warranting preventive attention, begins in midlife, not only in older age. Both daily physical activity and exercise were associated with lower long-term risk, and their combination provides the greatest benefit. Subjective functional assessment may represent a useful early intervention target.

**Supplementary Information:**

The online version contains supplementary material available at https://doi.org/10.1186/s11556-026-00424-y.

## Background

 Progressive deterioration of mobility represents a major global health challenge, as it is a key pathway leading to activity limitation, loss of independence, reduced social participation, and eventual need for long-term care in aging societies worldwide [1]. Importantly, mobility starts to decline earlier than is generally recognized, typically around the age of 40 years [2, 3]. Identifying modifiable factors that delay or prevent mobility decline before overt disability occurs is, therefore, a critical public health priority.

Locomotive syndrome (LS) is a clinical concept, initially proposed by the Japanese Orthopaedic Association, that describes reduced mobility caused by impairment of the musculoskeletal system, characterized by declines in walking ability, balance, and physical function. It focuses on detecting early mobility decline that precedes severe disability or dependence [4]. LS is evaluated using a combination of simple physical performance tests and a self-administered questionnaire, including the two-step test, the stand-up test, and a standardized questionnaire assessing difficulties in daily living [4]. Because these assessments are simple and feasible in large-scale health checkups, LS provides a practical framework for identifying individuals at risk of future mobility impairment [5]. Mobility decline evaluated using these tests has been shown to predict future care-need certification and represents a clinically meaningful decline in mobility function [6].

Previous longitudinal studies have demonstrated associations between physical activity and mobility-related outcomes [6–9]; however, several important gaps remain in relation to LS. Few studies have examined long-term incident LS using simple health checkup–based assessments of self-reported exercise habits and daily activity across different age groups [10, 11]. Although these indicators do not capture specific activity domains, they may be relevant when considering differences in feasibility and sustainability [12, 13]. In addition, previous studies have focused primarily on older adults or shorter follow-up periods, limiting age-specific understanding of cumulative LS risk across adulthood [6–9].

To address these gaps, longitudinal data with sufficient follow-up duration are needed to evaluate the cumulative risk of LS and to examine whether the simple physical activity evaluations, considered separately and in combination, are associated with incident LS across age groups. In this context, the present study utilized an eight-year health checkup–based cohort to examine age-specific associations of exercise habits and daily physical activity with the cumulative incidence of LS. Age-specific risk patterns may inform targeted prevention strategies by aligning behavioral guidance with periods when mobility decline begins to accelerate.

Therefore, this study aimed to investigate age-specific associations of exercise habits and daily physical activity with the cumulative incidence of LS over eight years in a large health checkup cohort.

## Methods

### Study design and data source

This retrospective cohort study analyzed data from a large-scale database systematically compiled from all individuals who underwent annual health checkups at a private clinic located in Saitama City, Japan [14]. Saitama City is an urban municipality with an estimated population of approximately 1.34 million as of 2024 [15], where tertiary industries, including the service and retail sectors, constitute the major economic activities [16]. The database includes demographic characteristics, medical history, medication use, lifestyle factors, and laboratory test results. In Japan, all full-time employees are legally required to undergo an annual health checkup, and employers are responsible for ensuring compliance. In addition to standard health checkup items, all participants at the clinic underwent LS risk tests as part of routine examinations.

### Participants

Individuals who received a health checkup at baseline (i.e., 2016) were eligible for inclusion. Participants were excluded if they had missing data on any component of the LS risk tests, missing information on exercise habits or daily physical activity, were classified as having LS at baseline, or did not attend any health checkups from 2017 to 2024. Accordingly, inclusion required LS risk test data at baseline and at least one follow-up assessment.

This study was approved by the Institutional Review Board of our institute (approval number: 957). Owing to the retrospective nature of the study, the requirement for written informed consent was waived. An opt-out approach was used for data disclosure, and all participant data were anonymized to ensure confidentiality.

### LS risk tests

Participants underwent LS risk tests at every clinic visit between 2016 and 2024, including the two-step test, the stand-up test, and the 25-item Geriatric Locomotive Function Scale (GLFS-25) [4]. In the two-step test, participants were instructed to take two maximal steps forward from a standing position without losing balance. The two-step test value was calculated by dividing the total length of the two steps by the participant’s height. Higher values indicate better mobility function. In the stand-up test, participants were asked to stand up from a seated position using one or both legs and maintain the standing posture for three seconds. Originally, the test is performed using chairs of varying heights (40, 30, 20, and 10 cm), with difficulty ranging from standing up with both legs from a 40-cm chair (easiest) to standing up with one leg from a 10-cm chair (most difficult). However, due to time constraints during routine health checkups, only the minimum tasks required for LS stage determination—standing on one leg from a 40-cm chair and standing on both legs from a 20-cm chair—were administered. The GLFS-25 is a self-administered questionnaire comprising 25 items: 16 related to activities of daily living, 4 to pain, 3 to social function, and 2 to mental health [17]. Each item is rated on a 5-point Likert scale ranging from 0 (no impairment) to 4 (severe impairment). The total score ranges from 0 to 100, with higher scores indicating poorer locomotive function.

The presence of LS was defined according to predefined thresholds for each assessment. Participants were classified as having LS if they met at least one of the following criteria: a two-step test value < 1.3, inability to stand up on one leg from a 40-cm chair in the stand-up test, or a GLFS-25 score ≥ 7 [4].

### Exercise habits and daily physical activity

At baseline (i.e., 2016), exercise habits were assessed using a question asking whether participants had engaged in light-to-moderate physical exercise that induced mild sweating for at least 30 min per session, at least twice per week, for at least one year. Daily physical activity was assessed using a question asking whether participants performed walking or an equivalent level of physical activity for at least one hour per day in their daily life. Participants responded to these questions with dichotomous answers (yes/no). These questionnaires have been widely used as evidence-based items in the Japanese Specific Health Checkup program [18].

Participants were classified into four groups according to baseline exercise habits and daily activity: Ex−/Da−, no exercise habits and no daily activity; Ex−/Da+, no exercise habits with sufficient daily activity; Ex+/Da−, exercise habits without sufficient daily activity; and Ex+/Da+, exercise habits with sufficient daily activity, consistent with previous Japanese Health Checkup-based studies [19]. This classification allows separate and joint evaluation of exercise and daily physical activity [12].

### Participant characteristics and lifestyle-related factors

In 2016, participants underwent anthropometric measurements and completed standardized questionnaires, which collected information on age, sex, lifestyle factors, and medical history [18]. Blood tests were performed to assess lifestyle-related diseases [18]. Anthropometric measurements included height, weight, and body mass index. The presence of metabolic syndrome was evaluated based on waist circumference, hypertension, diabetes mellitus, hypertriglyceridemia, and low high-density lipoprotein cholesterol levels, using blood test results and medication history. These conditions were assessed according to the criteria of the National Cholesterol Education Program Adult Treatment Panel III [20]. For fasting plasma glucose, an updated threshold of ≥ 100 mg/dL was applied instead of the original ≥ 110 mg/dL specified in the initial ATP III criteria, in accordance with subsequent revisions [21]. Waist circumference thresholds were modified for the Asian population: females ≥ 80 cm and males ≥ 90 cm [22]. The presence of anemia, which has been reported to be associated with LS, was also recorded [3]. In addition, participants completed questionnaires regarding lifestyle factors, including smoking status, alcohol consumption, sleep, and dietary habits (Supplement 1) [18].

### Statistical analysis

All analyses were conducted separately within three age groups (≤ 39 years, 40–64 years, and ≥ 65 years) to examine age-specific associations between physical activity patterns and the incidence of LS [11]. These categories were defined to reflect three life stages: younger adulthood, midlife, and older adulthood. The cutoff at 40 years was based on previous research suggesting that mobility decline and LS become apparent around this age [2, 3], whereas 65 years is a conventional threshold for older adults in epidemiologic studies. Baseline participant characteristics were summarized using descriptive statistics. Because most continuous variables did not follow a normal distribution, as confirmed by the Kolmogorov–Smirnov test, these variables were expressed as medians with interquartile ranges. Categorical variables were presented as numbers and percentages.

Within each age group, baseline characteristics were compared across the four physical activity groups (Ex−/Da−, Ex−/Da+, Ex+/Da−, and Ex+/Da +) using the Kruskal–Wallis test for continuous variables and the chi-square test for categorical variables.

The cumulative incidence of LS over 8 years (between 2016 and 2024) was estimated using the Kaplan–Meier method and compared among the four physical activity groups using the log-rank test. Time-to-event was calculated from the 2016 baseline assessment to incident LS or the last available LS assessment, with participants without incident LS censored at their last assessment. Because the database did not record reasons for discontinuation of follow-up, losses to follow-up may have included death and other reasons. Furthermore, the same analyses were conducted using the LS incidence defined separately by the two-step test, stand-up test, and GLFS-25. The associations between physical activity group and incident LS were further evaluated using Cox proportional hazards regression models. Hazard ratios and 95% confidence intervals were calculated for each group, with the Ex−/Da− group used as the reference category. Two multivariable models were constructed: Model 1 was adjusted for sex and age (as a continuous variable within each age stratum); Model 2 was further adjusted for body mass index, metabolic syndrome, anemia, smoking status, alcohol consumption, dietary habits, and sleep characteristics at baseline. Missing data in covariates were minimal (Supplement 2). Therefore, no imputation was performed, and observations with missing values in model variables were excluded from the corresponding Cox regression analyses. The proportional hazards assumption was assessed by visual inspection of log-minus-log survival plots within each age-stratified model. The survival plots were approximately parallel, indicating no major violation.

Furthermore, to assess whether the association between physical activity group and incident LS differed by age group, we fitted a Cox proportional hazards model in the overall cohort, including age group, physical activity group, and their interaction term. This pooled model was adjusted for the same covariates as Model 2. The overall statistical significance of the interaction between age group and physical activity group was evaluated using a Wald test. To assess the impact of exclusions on selection bias, baseline characteristics were compared between participants included in the analysis and those excluded.

A *P* value < 0.05 was considered statistically significant. Statistical analyses were performed using commercial statistical software (JMP Student Edition, JMP Statistical Discovery, Cary, NC, United States). No sample size calculation was performed prior to the study, as this was an observational cohort study using all eligible participants with available data during the study period. This study was reported in accordance with the STROBE statement for observational studies.

## Results

**Fig. 1:**
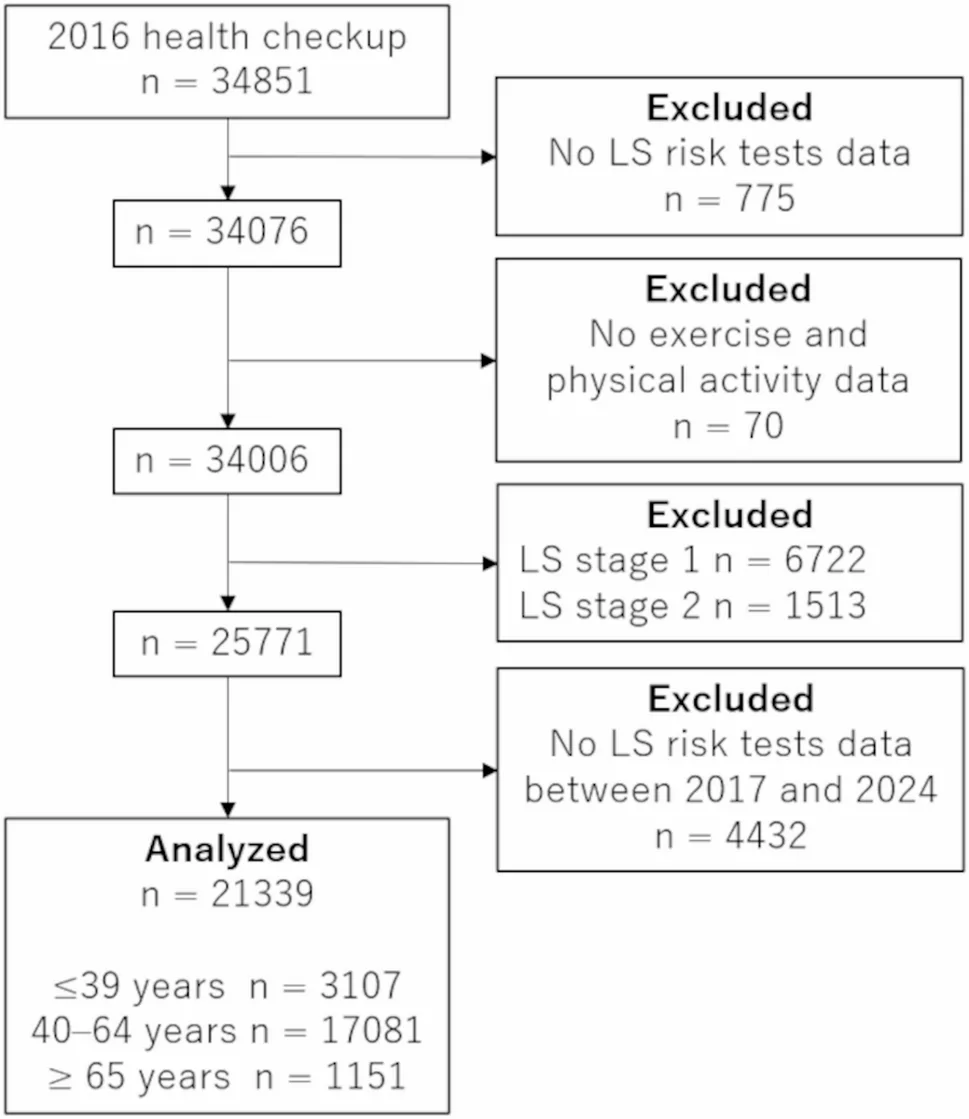
Participant flow

Of 34,851 individuals who underwent the baseline 2016 health checkup, 21,339 were included in the analysis after exclusions for missing baseline LS risk test data (*n* = 775), missing baseline exercise and/or daily physical activity data (*n* = 70), prevalent LS at baseline (*n* = 8255), and absence of follow-up LS risk test data (*n* = 4432) (Fig. 1). The median age was 48 (range, 21–84, interquartile value, 42, 56) years, and 8468 (40%) were female. At baseline, 11,252 participants (53%) were classified as Ex−/Da−, 5113 (24%) as Ex−/Da+, 1837 (9%) as Ex+/Da−, and 3137 (15%) as Ex+/Da+. Participants were stratified into three age groups: ≤ 39 years (*n* = 3107), 40–64 years (*n* = 17081), and ≥ 65 years (*n* = 1151) (Table 1).

**Table 1:** Baseline participant characteristics

	≤ 39 years (*n* = 3107)	40–64 years (*n* = 17081)	≥ 65 years (*n* = 1151)
Sex, female	1315 (42)	6783 (39)	370 (40)
Age, years^1^	37 (35, 38)	50 (45, 56)	68 (66, 70)
Height, m^1^	1.68 (1.61, 1.74)	1.66 (1.59, 1.72)	1.63 (1.56, 1.68)
Weight, kg^1^	61.9 (52.6, 71.2)	63.0(54.3, 71.6)	60.0 (52.4, 66.8)
Body mass index, kg/m^2^	21.9 (19.9, 24.2)	22.7 (20.6, 24.9)	22.5 (20.8, 24.4)
Metabolic syndrome	126 (4)	2284 (13)	328 (29)
Waist circumference, cm^1^	76.5 (71.0, 83.5)	79.5 (73.5, 85.0)	80.0 (75.0, 85.0)
High waist circumference	478 (15)	3835 (22)	231 (20)
Hypertension	426 (14)	6123 (36)	702 (61)
Diabetes	441 (14)	5495 (32)	563 (49)
High triglycerides	392 (13)	3851 (23)	416 (36)
High HDL cholesterol	181 (6)	2222 (13)	337 (29)
Anemia	125 (4)	967 (6)	33 (3)
Smoking	644 (21)	3260 (19)	108 (9)
Alcohol			
Rarely	1083 (35)	5613 (33)	427 (37)
Sometimes	1653 (53)	7879 (46)	414 (36)
Every day	371 (12)	3585 (21)	310 (27)
Insufficient sleep	1084 (35)	5842 (34)	185 (16)
Skip breakfast	755 (24)	2536 (15)	35 (3)
Late supper	1277 (41)	5678 (33)	135 (12)
Eat quicker	1345 (43)	7439 (44)	482 (42)
Two-step test^1^	1.49 (1.42, 1.56)	1.47 (1.40, 1.54)	1.44 (1.38, 1.50)
GLFS-25, points^1^	1 (0, 3)	2 (0, 3)	2 (0, 3)

Values show numbers and percentages unless indicated otherwise

^1^Median (25th, 75th quartiles). The results of the stand-up test were not presented because all participants demonstrated the same outcome, namely, the ability to stand up from a 40-cm chair with one leg

In participants aged ≤ 39 years and 40–64 years, more than half belonged to the inactive Ex−/Da− group, whereas in those aged ≥ 65 years, approximately 40% reported both habitual exercise and daily physical activity, being classified into the Ex+/Da+ group (Tables 2, 3 and 4). Across all age groups, cardiometabolic risk factors and lifestyle characteristics showed statistically significant differences among the four groups, with generally more favorable profiles observed in participants engaging in exercise and daily physical activity. Excluded participants had worse baseline LS risk test results than the included participants (Supplement 2); this was expected because the excluded group included individuals who already had LS at baseline and were therefore excluded by design. In addition, excluded participants were older, more often physically inactive, and had less favorable lifestyle profiles (Table 5).

**Table 2:** Exercise and daily activity of participants aged ≤ 39 years (*n* = 3107)

	Ex−/Da− (*n* = 1768, 57%)	Ex−/Da+ (*n* = 949, 31%)	Ex+/Da− (*n* = 141, 5%)	Ex+/Da+ (*n* = 249, 8%)	*P*
Sex, female	805 (46)	412 (43)	31 (22)	67 (27)	< 0.001
Age, years^1^	37 (35, 38)	37 (35, 38)	37 (36, 38)	37 (35, 38)	0.04
Height, m^1^	1.67 (1.60, 1.73)	1.67 (1.60, 1.74)	1.71 (1.65, 1.76)	1.70 (1.64, 1.75)	< 0.001
Weight, kg^1^	61.4 (51.9, 71.1)	61.3 (52.6, 70.2)	66.2 (57.3, 75.8)	64.7 (56.2, 73.4)	< 0.001
Body mass index, kg/m^2^	21.8 (19.8, 24.2)	21.8 (19.7, 24.2)	22.4 (20.8, 24.8)	22.3 (20.3, 24.4)	0.009
Metabolic syndrome	110 (6)	43 (5)	9 (6)	7 (3)	0.07
Waist circumference, cm^1^	76.5 (71.0, 83.5)	76.5 (71.0, 83.0)	77.0 (73.0, 83.5)	75.0 (70.5, 83.0)	0.31
High waist circumference	290 (16)	148 (16)	16 (11)	24 (10)	0.02
Hypertension	239 (14)	123 (13)	31 (22)	33 (13)	0.03
Diabetes	252 (14)	121 (13)	25 (18)	43 (17)	0.17
High triglycerides	235 (13)	119 (13)	17 (12)	21 (8)	0.19
High HDL cholesterol	112 (6)	52 (5)	6 (4)	11 (4)	0.46
Anemia	73 (4)	44 (5)	0 (0)	8 (3)	0.06
Smoking	378 (21)	194 (20)	31 (22)	41 (16)	0.33
Alcohol					
Rarely	641 (36)	333 (35)	37 (26)	72 (29)	0.06
Sometimes	931 (53)	496 (52)	86 (61)	140 (56)	
Every day	196 (11)	120 (13)	18 (13)	37 (15)	
Insufficient sleep	650 (37)	338 (36)	38 (27)	58 (23)	< 0.001
Skip breakfast	441 (25)	232 (24)	28 (20)	54 (22)	0.42
Late supper	721 (41)	389 (41)	67 (48)	100 (40)	0.46
Eat quicker	721 (41)	431 (45)	73 (52)	120 (48)	0.02
Two-step test^1^	1.48 (1.41, 1.56)	1.49 (1.42, 1.56)	1.51 (1.45, 1.59)	1.50 (1.43, 1.58)	0.001
GLFS-25, points^1^	1 (0, 3)	1 (0, 3)	1 (0, 3)	1 (0, 3)	0.43

Values show numbers and percentages unless indicated otherwise

^1^Median (25th, 75th quartiles). The results of the stand-up test were not presented because all participants demonstrated the same outcome, namely, the ability to stand up from a 40-cm chair with one leg

**Table 3:** Exercise and daily activity of participants aged 40–64 years (*n* = 17081)

	Ex−/Da− (*n* = 9121, 53%)	Ex−/Da+ (*n* = 3986, 23%)	Ex+/Da− (*n* = 1537, 9%)	Ex+/Da+ (*n* = 2437, 14%)	*P*
Sex, female	3595 (39)	1767 (44)	489 (32)	932 (38)	< 0.001
Age, years^1^	49 (44, 55)	49 (44, 55)	52 (46, 58)	52 (46, 57)	< 0.001
Height, m^1^	1.66 (1.59, 1.72)	1.65 (1.59, 1.72)	1.68 (1.61, 1.73)	1.66 (1.60, 1.72)	< 0.001
Weight, kg^1^	63.5 (54.6, 72.0)	61.8 (53.1, 70.6)	64.1 (55.8, 72.3)	62.9 (54.0, 70.9)	< 0.001
Body mass index, kg/m^2^	22.8 (20.7, 25.1)	22.4 (20.4, 24.7)	22.7 (20.9, 24.8)	22.5 (20.5, 24.6)	< 0.001
Metabolic syndrome	1658 (18)	665 (17)	287 (19)	399 (16)	0.04
Waist circumference, cm^1^	80.0 (74.0, 86.0)	79.0 (73.0, 85.0)	79.5 (74.5, 84.5)	78.0 (72.5, 83.5)	< 0.001
High waist circumference	2200 (24)	892 (22)	308 (20)	435 (18)	< 0.001
Hypertension	3206 (35)	1397 (35)	591 (38)	929 (38)	0.005
Diabetes	2923 (32)	1199 (30)	554 (36)	819 (34)	< 0.001
High triglycerides	2147 (24)	837 (21)	367 (24)	500 (21)	< 0.001
High HDL cholesterol	1192 (13)	499 (13)	219 (14)	312 (13)	0.38
Anemia	516 (6)	245 (6)	78 (5)	128 (5)	0.32
Smoking	1850 (20)	801 (20)	248 (16)	361 (15)	< 0.001
Alcohol					
Rarely	3101 (34)	1369 (34)	417 (27)	726 (30)	< 0.001
Sometimes	4130 (45)	1816(46)	756 (49)	1177 (48)	
Every day	1887 (21)	800(20)	364 (24)	534 (22)	
Insufficient sleep	3444 (38)	1351 (34)	456 (30)	591 (24)	< 0.001
Skip breakfast	1511 (17)	597 (15)	181 (12)	247 (10)	< 0.001
Late supper	3144 (34)	1343 (34)	461 (30)	730 (30)	< 0.001
Eat quicker	3765 (41)	1737 (44)	753 (49)	1184 (49)	< 0.001
Two-step test^1^	1.46 (1.40, 1.53)	1.47 (1.40, 1.54)	1.47 (1.41, 1.55)	1.47 (1.41, 1.54)	< 0.001
GLFS-25, points^1^	2 (0, 3)	1 (0, 3)	1 (0, 3)	1 (0, 3)	< 0.001

Values show numbers and percentages unless indicated otherwise

^1^Median (25th, 75th quartiles). The results of the stand-up test were not presented because all participants demonstrated the same outcome, namely, the ability to stand up from a 40-cm chair with one leg

**Table 4:** Exercise and daily activity of participants aged ≥ 65 years (*n* = 1151)

	Ex−/Da− (*n* = 363, 32%)	Ex−/Da+ (*n* = 178, 15%)	Ex+/Da− (*n* = 159, 14%)	Ex+/Da+ (*n* = 451, 39%)	*P*
Sex, female	110 (30)	55 (31)	56 (35)	149 (33)	0.67
Age, years^1^	67 (66, 70)	67 (66, 69)	67 (66, 69)	68 (66, 70)	< 0.001
Height, m^1^	1.64 (1.57, 1.69)	1.63 (1.56, 1.68)	1.63 (1.56, 1.68)	1.63 (1.56, 1.68)	0.31
Weight, kg^1^	61.0 (54.0, 68.3)	60.5 (50.8, 66.8)	60.5 (53.1, 67.5)	58.9 (51.4, 65.1)	0.03
Body mass index, kg/m^2^	22.7 (21.0, 24.7)	22.5 (20.9, 24.6)	22.7 (20.9, 24.8)	22.3 (20.7, 24.0)	0.04
Metabolic syndrome	138 (38)	58 (33)	61 (38)	136 (30)	0.07
Waist circumference, cm^1^	81.5 (76.5, 86.0)	80.5 (75.0, 86.0)	81.0 (76.0, 85.5)	79.0 (74.0, 83.0)	< 0.001
High waist circumference	89 (25)	26 (20)	35 (22)	71 (16)	0.02
Hypertension	219 (60)	105 (59)	102 (64)	276 (61)	0.79
Diabetes	178 (49)	77 (43)	73 (46)	235 (52)	0.20
High triglycerides	153 (42)	63 (35)	61 (38)	139 (31)	0.009
High HDL cholesterol	116 (32)	54 (30)	50 (31)	117 (26)	0.25
Anemia	13 (4)	4 (2)	5 (3)	11 (2)	0.75
Smoking	61 (17)	14 (8)	8 (5)	25 (6)	< 0.001
Alcohol					
Rarely	140 (39)	70 (39)	50 (31)	167 (37)	0.45
Sometimes	120 (33)	63 (35)	69 (43)	162 (36)	
Every day	103 (28)	45 (25)	40 (25)	122 (27)	
Insufficient sleep	76 (21)	29 (16)	32 (20)	48 (11)	< 0.001
Skip breakfast	15 (4)	6 (3)	3 (2)	11 (2)	0.42
Late supper	46 (13)	23 (13)	15 (9)	51 (11)	0.70
Eat quicker	142 (39)	72 (40)	75 (47)	193 (43)	0.35
Two-step test^1^	1.44 (1.38, 1.50)	1.42 (1.37, 1.49)	1.44 (1.38, 1.50)	1.44 (1.37, 1.51)	0.34
GLFS-25, points^1^	2 (1, 4)	2 (0, 4)	2 (1, 4)	1 (0, 3)	< 0.001

Values show numbers and percentages unless indicated otherwise

^1^Median (25th, 75th quartiles). The results of the stand-up test were not presented because all participants demonstrated the same outcome, namely, the ability to stand up from a 40-cm chair with one leg

**Table 5:** Incidence of locomotive syndrome and follow-up time

Age	Exercise and daily activity	Incident locomotive syndrome^1^	Follow-up time, years^2^	Cumulative incidence at 8 years
≤ 39 years	Ex−/Da− (*n* = 1768)	445 (25)	5 (2, 8)	0.30
(*n* = 3107)	Ex−/Da+ (*n* = 949)	220 (23)	5 (3, 8)	0.29
	Ex+/Da− (*n* = 141)	30 (21)	6 (3, 8)	0.25
	Ex+/Da+ (*n* = 249)	45 (18)	6 (3, 8)	0.23
40–64 years	Ex−/Da− (*n* = 9121)	3367 (37)	4 (2, 8)	0.45
(*n* = 17081)	Ex−/Da+ (*n* = 3986)	1367 (34)	4 (2, 8)	0.44
	Ex+/Da− (*n* = 1537)	490 (32)	5 (2, 8)	0.40
	Ex+/Da+ (*n* = 2437)	713 (29)	5 (2, 8)	0.37
≥ 65 years	Ex−/Da− (*n* = 363)	183 (50)	3 (1, 5)	0.71
(*n* = 1151)	Ex−/Da+ (*n* = 178)	85 (48)	3 (2, 6)	0.68
	Ex+/Da− (*n* = 159)	69 (43)	3 (2, 6)	0.69
	Ex+/Da+ (*n* = 451)	169 (37)	3 (2, 6)	0.55

^1^Number (%)

^2^Median (25th, 75th quartiles)

Overall, 7183 (34%) incident LS events occurred during follow-up, including 740, 5937, and 506 participants in the ≤ 39-year, 40–64-year, and ≥ 65-year groups, respectively (Table 5). In the ≤ 39-year group, cumulative LS incidence decreased monotonically from 0.30 in the inactive group (Ex−/Da−) to 0.23 in the most active group (Ex+/Da+) at eight years. In the 40–64-year group, cumulative incidence showed a similar stepwise pattern, decreasing from 0.45 in Ex−/Da − to 0.37 in Ex+/Da+. In the ≥ 65-year group, it was highest in Ex−/Da− (0.71) and lowest in Ex+/Da+ (0.55).

**Fig. 2:**
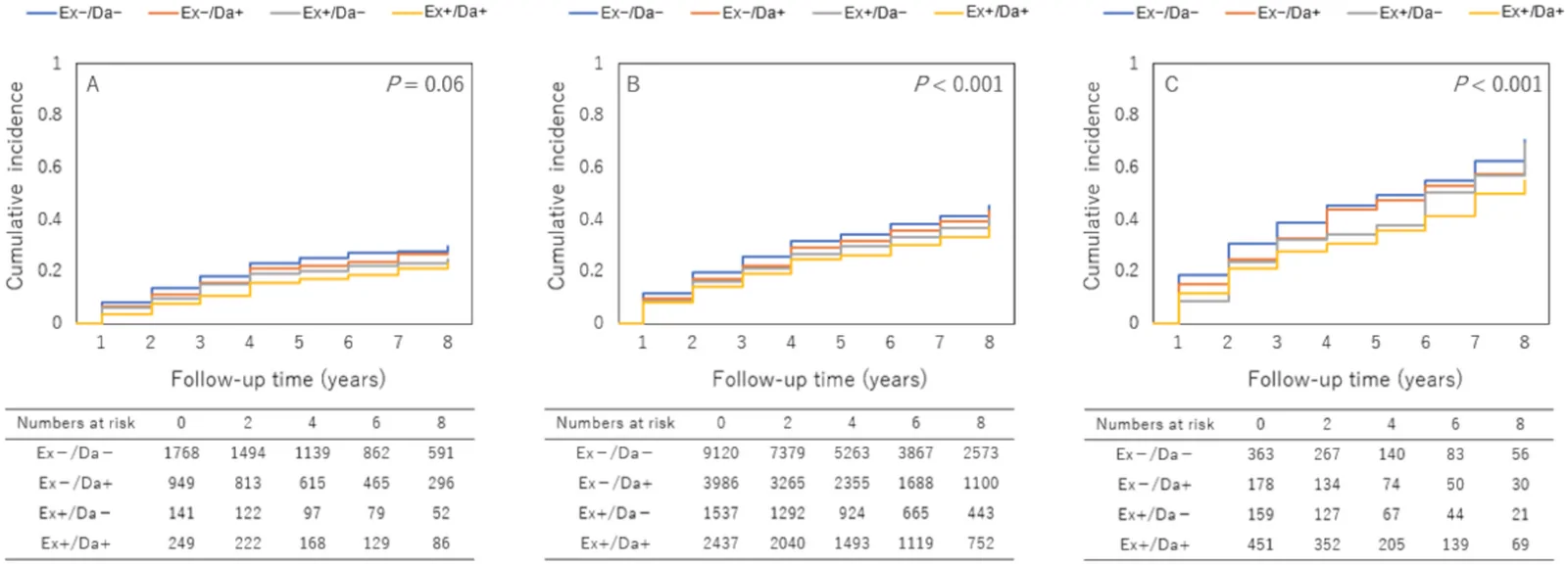
Cumulative incidence of locomotive syndrome. **A**, ≤ 39 years; **B**, 40–64 years; **C**, ≥ 65 years

In participants aged ≤ 39 years, separation of the cumulative incidence curves across the four exposure groups was modest and did not reach statistical significance (log-rank *P* = 0.06) (Fig. 2A). In contrast, statistically significant differences were observed in participants aged 40–64 years and ≥ 65 years (both *P* < 0.001), with progressively clearer separation of curves in older age strata (Fig. 2B and C). Across all age groups, the qualitative ordering of LS risk was highest in the Ex−/Da− group, intermediate in the Ex−/Da + and Ex+/Da− groups, and lowest in the Ex+/Da+ group. Kaplan–Meier curves for LS incidence, defined separately by the two-step test, stand-up test, and GLFS-25, showed that cumulative incidence increased with advancing age across all definitions (Supplement 3–5). Participants reporting both exercise and daily physical activity consistently had the lowest incidence, with this pattern most clearly evident when LS was defined using the GLFS-25. The cumulative incidence appeared to be higher for the GLFS-25 than for the two physical tests.

In Cox proportional hazards models using Ex−/Da − as the reference group, the overall association between physical activity group and incident LS was not statistically significant in participants aged ≤ 39 years in either Model 1 (*P* = 0.07) or Model 2 (*P* = 0.22) (Table 6). In contrast, statistically significant associations were observed in participants aged 40–64 years in both Model 1 and Model 2 (both *P* < 0.001), with the lowest hazard ratios observed in the Ex+/Da+ group. In participants aged ≥ 65 years, the overall association was also statistically significant in both Model 1 (*P* < 0.001) and Model 2 (*P* = 0.008), and the Ex+/Da+ group again showed the lowest hazard ratios. In the Cox model using the overall cohort, no statistically significant interaction was observed between age group and physical activity group (*P* for interaction = 0.99), suggesting that the magnitude of association did not differ across age groups.

**Table 6:** Cox proportional hazards regression analysis of incident locomotive syndrome according to exercise habits and daily physical activity

		Model 1		Model 2	
Age	Exercise and daily activity	Hazard ratio (95% confidence interval)	*P* ^1^	Hazard ratio (95% confidence interval)	*P* ^1^
≤ 39 years	Ex−/Da−	Ref	0.07	Ref	0.22
	Ex−/Da+	0.92 (0.78–1.09)		0.93 (0.79–1.09)	
	Ex+/Da−	0.79 (0.55–1.15)		0.80 (0.55–1.17)	
	Ex+/Da+	0.70 (0.51–0.95)		0.77 (0.56–1.05)	
40–64 years	Ex−/Da−	Ref	< 0.001	Ref	< 0.001
	Ex−/Da+	0.91 (0.86–0.97)		0.93 (0.88–0.99)	
	Ex+/Da−	0.81 (0.74–0.89)		0.86 (0.78–0.95)	
	Ex+/Da+	0.72 (0.67–0.79)		0.79 (0.72–0.85)	
≥ 65 years	Ex−/Da−	Ref	< 0.001	Ref	0.008
	Ex−/Da+	0.87 (0.67–1.13)		0.93 (0.72–1.21)	
	Ex+/Da−	0.80 (0.61–1.06)		0.81 (0.61–1.08)	
	Ex+/Da+	0.64 (0.52–0.79)		0.70 (0.56–0.86)	

Model 1: Adjusted for age and sex

Model 2: Adjusted for age, sex, body mass index, metabolic syndrome, anemia, smoking, alcohol, sleep, and diet

^1^*P* for overall association (likelihood ratio test)

## Discussion

In this large health checkup–based cohort with up to eight years of follow-up, we identified age-specific associations between patterns of exercise habits, daily physical activity, and the cumulative incidence of LS. Participants who engaged in both habitual exercise and daily activity consistently showed the lowest incidence of LS, particularly in middle-aged and older adults. These findings indicate that simple indicators of self-reported exercise habit and daily activity are associated with long-term risk of mobility decline. LS reflects early mobility impairments closely related to frailty and physical disability. Thus, the present findings clarify how these brief health checkup–based measures relate to early mobility changes.

### Principal findings

Across all age strata, a consistent gradient of risk was observed. Participants with neither exercise habits nor daily activity had the highest cumulative incidence of LS, those engaging in either behavior showed intermediate risk, and those engaging in both had the lowest risk. This pattern suggests that these associations persisted after adjustment for baseline lifestyle-related factors, suggesting that mobility decline is influenced by the cumulative effects of exercise and habitual movement in daily life [23, 24]. Such results are consistent with longitudinal perspectives that emphasize the interaction between behavior, functional reserve, and environmental demands over time.

From a clinical perspective, these findings may help identify individuals with physical activity as a pragmatic target for early advice and monitoring in routine health checkups, particularly in middle-aged and older adults. When structured exercise is difficult to implement, consciously increasing movement in daily routines—such as walking during commuting, using stairs, and moving during work or household tasks—may contribute to preserving mobility [23, 25]. Conversely, when opportunities for daily movement are limited, allocating time for habitual exercise—such as scheduling regular walks before or after work, performing brief home-based exercise sessions on otherwise sedentary days, or attending a community exercise class—may partially compensate for the adverse consequences of inactivity [26, 27]. In this study, the lowest risk was consistently observed among individuals who reported both daily activity and exercise habit at baseline. However, this finding should be interpreted cautiously because it may reflect the benefit of a greater volume of physical activity overall rather than engagement in specific types of exercise.

### Age-specific implications

Age-stratified analyses revealed heterogeneity in the associations between activity patterns and LS incidence. In middle-aged adults (40–64 years), both daily physical activity and exercise independently contributed to lower risk after extensive adjustment. This finding highlights midlife as an important period during which behavioral exposures may be associated with later mobility outcomes [28, 29]. At this stage, relatively modest changes in movement behavior may still meaningfully alter long-term mobility trajectories [30, 31].

In older adults (≥ 65 years), only the combination of exercise and daily physical activity remained statistically significantly associated with reduced risk after full adjustment. This pattern may reflect a smaller sample size than the middle-aged group, as well as greater heterogeneity in health status and lower functional reserve, which can attenuate the apparent effects of either behavior alone [32, 33]. The direction of association, however, was consistent with that observed in middle-aged adults, with lower hazard ratios at higher activity levels, suggesting that maintaining mobility in later life may require engagement in multiple forms of movement.

In younger adults (≤ 39 years), associations were weak and attenuated after full adjustment, likely reflecting lower absolute risk and greater functional reserve, consistent with previous studies [3, 34]. Nevertheless, the directionality of associations was consistent with those observed in older age groups, supporting the notion that movement behaviors established earlier in adulthood may influence long-term mobility outcomes, even if their effects are not immediately apparent.

### Insights from LS risk test–specific analyses

The cumulative incidence of LS was highest when defined using the GLFS-25, followed by the stand-up test and the two-step test. This pattern suggests that self-reported functional difficulty, including perceived mobility limitation, pain-related restriction, and reduced participation in daily life, may capture earlier stages of mobility decline than objective performance measures, which tend to detect impairment at a later stage [35]. Clinically, patient-reported measures such as the GLFS-25 may complement performance-based assessments by providing insight into functional difficulties that are not yet apparent on objective testing.

Objective performance tests remain essential for assessing physical capacity, but the stand-up and two-step tests used in our study may be subject to ceiling effects in relatively healthy populations, particularly at younger ages. In addition, these tests primarily reflect maximal or near-maximal performance under standardized conditions and may be less sensitive to subtle functional constraints encountered in daily life. Nevertheless, the complementary use of subjective and objective measures, therefore, enhances the characterization of mobility decline and better captures its multidimensional nature across the life course [4].

Across all test-specific definitions, higher levels of physical activity were generally associated with lower cumulative incidence, consistent with the results of the overall LS assessment. These results indicate that the inverse association between activity level and LS incidence was robust and not dependent on the particular assessment method used. From a life-course perspective, these age-specific patterns are consistent with mobility decline being a progressive process across adulthood rather than a phenomenon confined to old age [2]. Younger adults may retain sufficient functional reserve, whereas in midlife and older age, differences in movement-related behaviors may be more clearly reflected in mobility-related outcomes [30].

### Strengths and limitations

Strengths of this study include the large sample size, extended follow-up duration, and repeated functional assessments embedded in routine health checkups. The use of survival analysis enabled explicit modeling of the time to incident mobility impairment over time. Separating exercise from daily physical activity enhanced translational relevance, as these behaviors differ in feasibility and sustainability.

Several limitations should be considered. Exercise habits and daily physical activity were assessed using brief self-reported binary items at a single baseline time point. Therefore, misclassification and changes in behavior during follow-up could not be excluded. Furthermore, dose–response relationships or specific activity domains could not be evaluated. The study population consisted primarily of individuals participating in regular health checkups in urban areas of Japan. Moreover, those excluded from the analysis had less favorable baseline health and lifestyle profiles. Therefore, the included participants may be more health-conscious than the general population, raising the possibility of healthy volunteer bias and limiting generalizability to rural settings or populations with different socioeconomic or cultural backgrounds. In addition, although extensive covariates were included in the multivariable models, unmeasured confounding cannot be fully excluded, including psychological factors, such as depressive symptoms and sarcopenia, and social inequality–related factors, such as financial circumstances and social support, which have been linked to LS and may also influence physical activity behaviors [36, 37]. Future studies should examine these factors and possible sex-specific differences in these associations. The database did not record the reasons for incomplete LS testing. Therefore, because non-completion may have reflected diverse reasons, including health-related concerns and personal choice, some bias in the estimated incidence and associations cannot be excluded. Finally, because LS was assessed at periodic health checkups, the exact onset time of incident LS was not directly observed and may have occurred between two assessments. The database also did not record reasons for discontinuation of follow-up. Therefore, death could not be identified separately as a competing event, and some bias in the estimated incidence and associations cannot be excluded.

## Conclusion

In conclusion, this eight-year longitudinal study shows that different patterns of exercise habits and daily physical activity are differentially associated with the cumulative incidence of mobility decline, as assessed by incident LS. Engaging in either daily physical activity or exercise at baseline was associated with lower risk, while their combination conferred the greatest benefit, particularly in middle-aged and older adults. These findings underscore the importance of promoting both an active daily lifestyle and habitual exercise to support long-term mobility across adulthood.

## Supplementary Information

Supplementary Material 1.

## Data Availability

The datasets generated and/or analyzed during the current study are not publicly available because the data originated from a single clinical site and are not open access. However, they are available from the corresponding author on reasonable request.

## Abbreviations

LS: Locomotive Syndrome
GLFS-25: 25-item Geriatric Locomotive Function Scale
