# Supplementary material for: Exercise, daily physical activity, and age-specific cumulative incidence of mobility decline: an 8-year cohort study of locomotive syndrome
Source: Eur Rev Aging Phys Act. 2026 Jun 29;23:34. doi: 10.1186/s11556-026-00424-y (PMC13595768; doi:10.1186/s11556-026-00424-y)
Supplement: Supplementary file 1 [file 11556_2026_424_MOESM1_ESM.docx]

**Supplement 1** Health checkup questionnaire

| Category | Questions | Answer options |
| --- | --- | --- |
| Medical history | Have you ever been diagnosed as anemic? | Yes / No |
|  | Are you taking medication to reduce blood pressure? | Yes / No |
|  | Are you taking medication to reduce blood sugar or insulin injections? | Yes / No |
|  | Are you taking medication to reduce your level of cholesterol or neutral fat? | Yes / No |
| Lifestyle | Are you currently a smoker? | Yes / No |
|  | How often do you drink? | Every day / Sometimes / Rarely |
|  | Do you sleep well and enough? | Yes / No |
| Diet | Is your eating speed quicker than others? | Quicker / Normal / Slower^a^ |
|  | Do you eat supper two hours before bedtime more than 3 times a week? | Yes / No |
|  | Do you skip breakfast more than 3 times a week? | Yes / No |

^a^Answers of “normal” and “slower” were combined for statistical analysis because of a low proportion of answers “slower.”

**Supplement 2** Baseline characteristics of participants included in and excluded from the analysis.

|  | | Included (n = 21339) | Excluded (n = 13512) | *P* |
| --- | --- | --- | --- | --- |
| Sex, female | | 8468 (40) | 6536 (48) | < 0.001 |
| Age, years^1^ | | 48 (42, 56) | 51 (43, 58) | < 0.001 |
| Height, m^1^ | | 1.66 (1.59, 1.72) | 1.64 (1.58, 1.71)^6^ | < 0.001 |
| Weight, kg^1^ | | 62.6 (53.9, 71.2) | 62.3 (53.5, 71.9)^6^ | 0.66 |
| Body mass index, kg/m^1^ | | 22.5 (20.5, 24.8) | 22.9 (20.7, 25.43) | < 0.001 |
| Metabolic syndrome | | 3571 (17) | 3085 (23) | < 0.001 |
|  | Waist circumference, cm^1^ | 79.0 (73.0, 85.0) | 80.5 (74.0, 87.5)^6^ | < 0.001 |
|  | High waist circumference | 4544 (21) | 4274 (32)^6^ | < 0.001 |
|  | Hypertension | 7251 (34) | 5263 (39)^3^ | < 0.001 |
|  | Diabetes | 6499 (30)^2^ | 4516 (33)^4^ | < 0.001 |
|  | High triglycerides | 4659 (22)^2^ | 3414 (25)^3^ | < 0.001 |
|  | High HDL cholesterol | 2740 (13)^2^ | 2216 (16)^3^ | < 0.001 |
| Anemia | | 1125 (5)^2^ | 949 (7)^7^ | < 0.001 |
| Smoking | | 4012 (19)^2^ | 2850 (21)^8^ | < 0.001 |
| Alcohol | |  |  | < 0.001 |
|  | Rarely | 7123 (33)^3^ | 5358 (40)^9^ |  |
|  | Sometimes | 9946 (47)^3^ | 5555 (41)^9^ |  |
|  | Every day | 4266 (20)^3^ | 2586 (19)^9^ |  |
| Insufficient sleep | | 7111 (33)^4^ | 6004 (45)^10^ | < 0.001 |
| Skip breakfast | | 3326 (16)^5^ | 2442 (18)^11^ | < 0.001 |
| Late supper | | 7090 (33)^5^ | 4556 (34)^12^ | 0.33 |
| Eat quicker | | 9266 (43)^5^ | 6092 (45)^11^ | 0.003 |
| Two-step test^1^ | | 1.47 (1.40, 1.54) | 1.43 (1.36, 1.51)^13^ | < 0.001 |
| GLFS-25, points^1^ | | 1 (0, 3) | 7 (2, 11)^14^ | < 0.001 |
| Physical activity | |  |  | < 0.001 |
|  | Ex−/Da−^1^ | 11252 (53) | 7620 (57)^15^ |  |
|  | Ex−/Da+^1^ | 5113 (24) | 3144 (23)^15^ |  |
|  | Ex+/Da−^1^ | 1837 (9) | 999 (7)^15^ |  |
|  | Ex+/Da+^1^ | 3137 (15) | 1710 (13)^15^ |  |

Values show numbers and percentages unless indicated otherwise. ^1^Median (25^th^, 75^th^ quartiles). Number of missing data: ^2^n=1, ^3^n=4, ^4^n=2, ^5^n=9, ^6^ n=3, ^7^n=7, ^8^n=6, ^9^n=13, ^10^n=25, ^11^n=10, ^12^n=11, ^13^n=768, ^14^n=34, ^15^n=39.

**Supplement 3** Cumulative incidence of locomotive syndrome assessed using the two-step test. A, ≤ 39 years; B, 40–64 years; C, ≥ 65 years

**Supplement 4** Cumulative incidence of locomotive syndrome assessed using the stand-up test. A, ≤ 39 years; B, 40–64 years; C, ≥ 65 years

**Supplement 5** Cumulative incidence of locomotive syndrome assessed using the 25-item Geriatric Locomotive Function Scale. A, ≤ 39 years; B, 40–64 years; C, ≥ 65 years
